# Assessment of Sieverts Law Assumptions and ‘*n*’ Values in Palladium Membranes: Experimental and Theoretical Analyses

**DOI:** 10.3390/membranes11100778

**Published:** 2021-10-12

**Authors:** Abdulrahman Alraeesi, Tracy Gardner

**Affiliations:** 1 Chemical and Petroleum Engineering Department, United Arab Emirates University, Al Ain P.O. Box 15551, United Arab Emirates; 2 Chemical and Biological Engineering Department, Colorado School of Mines, Golden, CO 80401, USA; tgardner@mines.edu

**Keywords:** concentration polarization, palladium foil, palladium-silver membrane, permeation, hydrogen permeation, mixed feed gas, Sieverts’ law, *n* value, transport modeling

## Abstract

Palladium and palladium alloy membranes are superior materials for hydrogen purification, removal, or reaction processes. Sieverts’ Law suggests that the flux of hydrogen through such membranes is proportional to the difference between the feed and permeate side partial pressures, each raised to the 0.5 power (*n* = 0.5). Sieverts’ Law is widely applied in analyzing the steady state hydrogen permeation through Pd-based membranes, even in some cases where the assumptions made in deriving Sieverts’ Law do not apply. Often permeation data are fit to the model allowing the pressure exponent (*n*) to vary. This study experimentally assessed the validity of Sieverts’ Law as hydrogen was separated from other gases and theoretically modelled the effects of pressure and temperature on the assumptions and hence the accuracy of the 0.5-power law even with pure hydrogen feed. Hydrogen fluxes through Pd and Pd-Ag alloy foils from feed mixtures (5–83% helium in hydrogen; 473–573 K; with and without a sweep gas) were measured to study the effect of concentration polarization (CP) on hydrogen permeance and the applicability of Sieverts’ Law under such conditions. Concentration polarization was found to dominate hydrogen transport under some experimental conditions, particularly when feed concentrations of hydrogen were low. All mixture feed experiments showed deviation from Sieverts’ Law. For example, the hydrogen flux through Pd foil was found to be proportional to the partial pressure difference (*n* ≈ 1) rather than being proportional to the difference in the square root of the partial pressures (*n* = 0.5), as suggested by Sieverts’ Law, indicating the high degree of concentration polarization. A theoretical model accounting for Langmuir adsorption with temperature dependent adsorption equilibrium coefficient was made and used to assess the effect of varying feed pressure from 1–136 atm at fixed temperature, and of varying temperature from 298 to 1273 K at fixed pressure. Adsorption effects, which dominate at high pressure and at low temperature, result in pressure exponents (*n*) values less than 0.5. With better understanding of the transport steps, a qualitative analysis of literature (*n*) values of 0.5, 0.5 < *n* < 1, and *n* > 1, was conducted suggesting the role of each condition or step on the hydrogen transport based on the empirically fit exponent value.

## 1. Introduction

Pure hydrogen gas is considered to be a valued chemical product, due to its use in refinery processes and as a feedstock for ammonia synthesis [1,2]. Furthermore, in recent years, hydrogen has been valued as a promising form of energy storage for sporadic renewable power and as a clean fuel [3]. The conventional method for hydrogen production is primarily steam reforming of fossil fuels [4,5]. Historically, hydrogen is produced from fossil fuels, out of which 60% is from dedicated primary hydrogen-producing facilities. In the Middle East, hydrogen from natural gas (NG) costs (USD 1/kg H_2_). As far production is concerned, 71.27% of hydrogen is produced from NG, 27.27% from coal, and the remaining 1.4% is equally divided between petroleum and water electrolysis. Most recently, bio-hydrogen production has gained attention compared with fossil-based hydrogen. Biomass gasification of bio-wastes is commercially available with 88% energy conversion efficiency, while processes such as dark and photo fermentation, bio photolysis, and microbial electrolysis cell (MEC) are still under development, and the combined energy conversion efficiency of all processes does not exceed 25% [6]. The latter processes have some limitations: (i) the bio-photolysis requires an external light source and a customized bioreactor and still the hydrogen yield is low; (ii) the dark fermentation is thermodynamically limited and favors the pretreatment of certain wastes with the need of an additional separation unit for H_2_ purification plus the effluent has a high BOD; (iii) the photo-fermentation is only suitable for VFA-rich waste, constrained by day-night cycle (i.e., an external source of light) and produces low hydrogen rate with low light conversion efficiency; and (iv) MEC: despite the high H_2_ yield the production rate is low and there is a demand for external voltage as well as the catalyst [6,7]. The limitations can be accommodated and overcome by the use of: an efficient and properly designed bioreactor, appropriate process modifications, a suitable feedstocks, and an efficient microbial strains [8]. Moreover, because of the high H_2_ diffusivity, the low density (as gas or liquid), the higher gravimetric energy content and the wider flammability limits compared to most fossil fuels; hydrogen cost and safety obstacles are there at every step of distribution, shipment, or storage. Hydrogen can be delivered as a pressurized gas, or in the liquid form, combined in an absorbing metallic alloy matrix or transported in a chemical precursor form such as lithium. Hydrogen storage-classified risk is a concern, and hence an electrochemical hydrogen storage was developed by the integration of a solid multi-walled carbon nanotube (MWCNT) electrode in a modified unitized regenerative fuel cell (URFC). The developed porous MWCNT electrode had electrochemical hydrogen storage capacity of 2.47 wt%, comparable with commercially available AB5-based hydrogen storage [9,10,11].

Some industrial applications such as combustion are capable to use the reformate gas mixture with no treatment, while other applications need the feed gas to be highly purified in hydrogen [12] by use of a well-known technology as the pressure swing absorption (PSA) [13]. However, this method is still suffering from complexity and relevant high energy requirements. An attractive and suitable purification alternative technology for PSA, is the use of dense palladium (Pd) metal membranes. Palladium membranes are basically considered to be a passive system, where in such system, the driving force for full separation is basically the hydrogen partial pressure across the membrane [14]. Palladium-based membranes (Pd or Pd-alloy) are unique in their relative thermal stability and hence operates at high temperatures to save the capital and the operating cost through retaining both hydrogen and carbon dioxide at high pressures [15,16]. A hydrogen separation membrane unit can be ideally applicable in gas cleaning (upstream technology) and in fuel cells and/or hydrogen turbines (downstream technology) [16]. Additionally, membranes can be combined to catalysts in order to conduct simultaneously equilibrium-limited reactions and hydrogen separation in a single unit called catalytic membrane reactor (CMR) [17]. Consequently, Palladium membranes have been often used for water gas shift and steam reforming reactions to shift the reaction and achieve high conversion and hence promote the production of highly purified hydrogen [18]. However, some limitations in palladium membranes performance have been encountered due to incorporation of less expensive materials and moving to the composite structure, permeability evaluation of impure feed gas containing hydrogen molecule, and finally optimization of the operating conditions. Therefore, transport resistances and the assumptions used in deriving the Sieverts’ Law, the hydrogen flux-pressure dependence equation, to be carefully evaluated.

**Figure 1:**
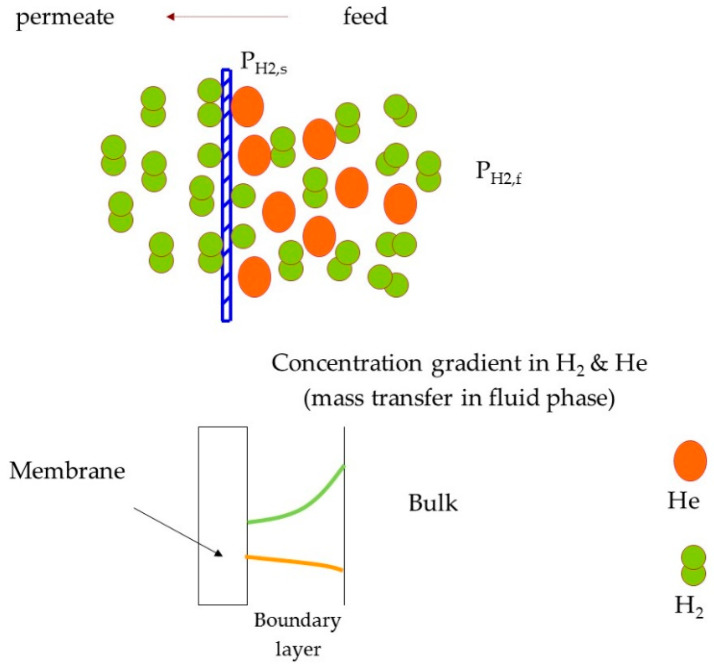
Schematic of hydrogen transport through a fluid phase on the feed side and then through the metal membrane.

Hydrogen transport in palladium-based membranes starts by the adsorption of hydrogen molecules on the membrane surface, followed by dissociation into atomic hydrogen, then transition of atomic hydrogen from the surface into the bulk metal, then atomic diffusion through the bulk metal, re-combinative desorption, and finally gas transport away from the surface to the bulk gas [19]. The existence of other gas components with hydrogen in the feed (as gas mixture) can decrease the permeation of hydrogen through the membrane [20,21]. The mixed feed, with fast hydrogen depletion in the membrane, develops a fluid phase resistance that either decreases the effective hydrogen partial pressure difference for permeation or limits the dissociative adsorption of hydrogen on the palladium surface [20]. There are several scenarios to describe the decrease of hydrogen-permeation (i) hydrogen dilution in the feed by the presence of other gas components, (ii) hydrogen depletion of the bulk feed due to hydrogen removal along the length of the membrane module, (iii) the build-up of a hydrogen-depleted layer adjacent to the membrane surface as a result of gas phase mass transport limitation, and possible competitive adsorption of mixture components on the membrane surface [22]. The Sieverts’ law can be applied to predict and evaluate the hydrogen permeation flux when Pd-H_2_ system considered as infinitely diluted or has “Ideal behavior”, in this scenario, the hydrogen diffusion in the lattice controls permeation and, at the same time, the H-concentration in the same lattice is so low [23]. It is worth mention that the previous described conditions are essential but not sufficient for pressure exponent to be 0.5. Overall, all metal membranes are far from ideal behavior. Therefore, major deviations from the Sieverts’ law have been noticed in the literature [24,25]. A main experimental finding for very thick palladium membranes, i.e., either thick Pd or ultrathin Pd composite membrane, is that the permeation behavior can considerably diverge from the Sieverts’ law [24,26], and it can be related to the solubility and diffusivity in the metal lattice [27,28,29]. Additionally, some theoretical case studies reported a different Sieverts’ pressure exponent n from the ideal value even in the presence of pure hydrogen feed [27,30,31]. Flanagan and Wang [32,33,34] presented a common approach to evaluate the Einstein hydrogen diffusivity (referred to as “intrinsic” or “ideal” diffusivity) and the nonideal correction from the experimental isotherms of the thermodynamic factor versus the atom ratio, in addition to the case where the excess of chemical potential is estimated by a linear function of the atom ratio. Another approach is proposed by Hara and coworkers, who established a methodology to assess the intrinsic concentration-independent hydrogen permeability and solubility by means of polynomial functions of the square root of the hydrogen partial pressure [27,35]. However, the continuous development and improvement of high-performing thin metal membranes is making the external mass transfer more and more significant and hence increase the complexity of the approach. Peters et al. (2008) [36] quantified the external mass transfer effect on hydrogen permeation through a 2.2 mm-thick Pd-alloy tubule membrane reactor system. A maximum H_2_ flux of 1223 mL cm^−2^ min^−1^ was obtained at 400 °C and 26 bar pure hydrogen feed pressure while in a mixture of 50% H_2_ + 50% N_2_ a maximum H_2_ flux of 230 mL cm^−2^ min^−1^ was at 26 bar. Even in water gas shift gas mixtures conditions a stronger influence of the dilution and the mass transfer, on the hydrogen flux, was largely observed. In general, the concentration polarization is a visible element in delaying hydrogen permeation [37,38]. Concentration polarization is generated by the stimulated concentration boundary layer along the membrane surface and this results in a lower hydrogen partial pressure at the retentate side of the membrane. The process of concentration polarization is schematically illustrated in Figure 1 across a dense palladium foil in the presence of hydrogen-helium feed mixture, where P_H2,s_ and P_H2,f_ are the partial pressure of hydrogen at the surface and in the bulk fluid, respectively.

According to this phenomena, hydrogen permeation deviates from Sieverts’ law and the model of continuous stirred tank reactor (CSTR) cannot be applied, particularly close to the membrane surface zone [39], hence, the mass transfer across the Pd membrane approaches the behavior of plug flow reactor (PFR). Concentration polarization may be intensified with low feed gas flow rate, high membrane permeability and selectivity, high H_2_ partial pressure difference across the membrane and at high membrane temperature [17,40]. In other words, increasing feed gas flow rate can significantly minimize the concentration polarization effect, as it flushes the fluid phase layer close to Pd surface. According to literature, the concentration polarization is a noticeable factor in retarding hydrogen permeation, where normally a pressure exponent (*n*) value in the range of 0.5–1.0 is reported [41,42,43]. If an appropriate pressure exponent is employed, the ratio of H_2_ flux and H_2_ partial pressure difference is characterized by a constant and one is able to identify the permeate of the tested membrane from the ratio. The pressure exponent (*n*), which is typically treated in the literature as an empirical fit parameter and normally an appropriate value is employed for analyzing permeation fluxes, differs from one study to another. In the current study some (*n*) values from literature, for a more complicated system with supported and unsupported Pd membranes in cases of pure and mixture feeds, are discussed.

Recall that the fast development of Pd-based membrane modules and the relevant applications of hydrogen purification/removal/catalytic reactions requires a better understanding of the hydrogen transport and the limitations in the Pd-based membrane structures as well as the high temperature and pressure operations. Therefore, the effect of operating conditions (T, P) on pressure exponent (*n*) was modeled, as well as the experimental easement of CP effect. It is worth to add here that despite the concentration polarization drawback of a lower flux, it can potentially be used as an advantage in the catalytic membrane reactor (CMR) system [44]. For example, in hydrogenation reaction, without altering the reaction pressure or temperature it is possible to adjust the feed composition and therefore lower the hydrogen penetration to the reaction zone. Controlling the hydrogen flux through the membrane would consequently controls the hydrogen coverage on the catalyst surface at permeate side, and subsequently, the selective hydrogenation reaction rate as well as reaction yield can be theoretically controlled [45,46,47]. Furthermore, the oxidation state of a catalyst deposited on the downstream side of a palladium-based membrane reactor could potentially be dynamically adjusted during reaction by adjusting the hydrogen feed composition or conditions [48,49,50]. For example, if the catalyst is reducing too quickly, the hydrogen flow rate could be reduced by diluting the hydrogen feed more. To model and analyze hydrogen permeation in such systems, the transport mechanism must be well understood.

The current work is a fundamental study on hydrogen permeation through palladium-based membranes to experimentally and theoretically assess the assumptions in deriving the Sieverts’ Law in cases of concentration polarization and diverse operating conditions. The findings of the study are used to evaluate and interpret the literature-based reported pressure exponent (*n*) values where it is considered an empirical fit parameter.

## 2. Theory

Hydrogen permeates through all dense metallic membranes including palladium-based membranes via the same mechanism as a complex multistep process [51,52]. The transport through these membranes is modeled using the solution-diffusion approach. In the solution-diffusion model, the flux of hydrogen gas through Pd-based membranes is the product of its solubility, a measure of the amount of hydrogen absorbed in the membrane under equilibrium conditions, and its diffusivity, a measure of how fast hydrogen atoms transport through the membrane [53,54,55].

**Figure 2:**
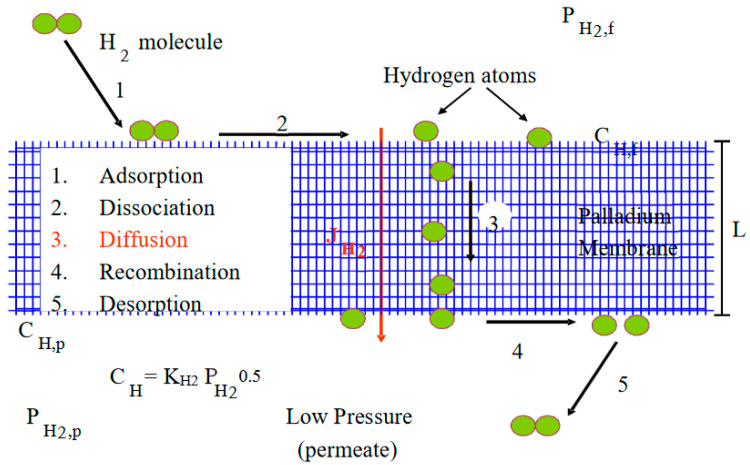
Schematic of hydrogen transport mechanism through Pd-based membranes.

Hydrogen permeation through palladium membranes is a complex, multi-step process, as shown in Figure 2.

Hydrogen molecules dissociatively adsorb on the surface (steps 1 and 2), diffuse through the bulk metal (step 3) as H atoms, and recombine as molecular hydrogen and desorb from the permeate side (steps 4 and 5). Assuming constant diffusivity in the bulk, step 3 is rate limiting, and that the driving force for transport can be represented by the sorbed phase atomic hydrogen gradient, Fick’s Law gives the steady state hydrogen flux, J_H2_ (mol/m^2^ s), as:

(1) $$J_{H2}=\frac{-DN_{b}}{2}\frac{dX}{dz}$$

where D is the hydrogen diffusivity in the bulk metal (m^2^/s), X is the bulk composition of hydrogen inside the membrane (mol H/mol Pd), N_b_ is the bulk metal Pd concentration (mol Pd/m^3^), and z is the spatial dimension along the thickness of the membrane. The 2 comes from the fact that there are 2 moles of H in one mole of H_2_. At steady state, the flux is independent of z, so dX/dz must also be constant (i.e., there is a linear bulk phase concentration profile). Thus Equation (1) can be re-written as:

(2) $$J_{H2}=\frac{D}{2L}N_{b}\left(X_{f}-X_{p}\right)$$

where X_f_ and X_p_ are the hydrogen compositions inside the membrane on the feed and permeate sides, respectively. The bulk hydrogen concentrations (X’s), in Equation (2) are related to the hydrogen pressure, P_H2_, by:

(3) $$X=X_{m}\theta =\frac{X_{m}\sqrt{\frac{K_{H2}}{RT}P_{H2}}}{1+\sqrt{\frac{K_{H2}}{RT}P_{H2}}}$$

where K_H2_ is the adsorption equilibrium constant and Ө is the fractional surface coverage of hydrogen. Assuming diffusion of atomic hydrogen through bulk metal to be the slowest step in the process and is considered to be rate limiting as per the Fickian diffusion, the broader steady state hydrogen permeation flux; J_H2_ (mol/m^2^ s), through palladium membranes [22,56,57,58] can be obtained by substituting Equation (3) into (2) as follows:

(4) $$J_{H2}=\frac{DN_{b}}{2L}\left(\frac{X_{m}\sqrt{\frac{K_{H2}}{RT}P_{H2,f}}}{1+\sqrt{\frac{K_{H2}}{RT}P_{H2}}}-\frac{X_{m}\sqrt{\frac{K_{H2}}{RT}P_{H2,p}}}{1+\sqrt{\frac{K_{H2}}{RT}P_{H2,p}}}\right)$$

where D is the diffusion coefficient (m^2^/s), L is the membrane thickness (m), K_H2_ is the adsorption equilibrium constants on feed or permeate side (mol/m^3^), and P_H2,f_ and P_H2,p_ are the feed and permeate side partial pressures of H_2_ (Pa), respectively.

In order to obtain the above equation, the following assumptions were employed:

- Diffusion of H through Pd metal is rate limiting (for membrane thickness ≥10 µm);
- Surface coverage of H at both the feed and permeate sides of the membrane is in equilibrium with the respective fluid phases;
- There is no concentration gradient at feed side (i.e., from bulk feed to membrane surface);
- Constant diffusion coefficient in Pd, i.e., D ≠ f (P_H2_);
- Linear H in metal phase concentration profile, i.e., Fick’s Law can be applied;
- No species other than H on surface—either adsorbed molecules or contaminants, i.e., palladium sites are not blocked and hence the effective area for permeation is the same throughout the process.

Furthermore, by assuming weak adsorption on surface, at low pressure and high temperature, we can get

$$\sqrt{\frac{K_{H2}}{\mathrm{RT}}P_{H2}}\ll 1$$

Moreover, if the adsorption equilibrium constant is assumed to be the same on both sides of the membrane, then the expression of Equation (4) can be simplified in the Sieverts’ Law format as follows

(5) $$J_{H2}=\frac{Q_{H2}}{L_{}}\left(P_{H2,f}^{0.5}-P_{H2,p}^{0.5}\right)$$

where Q_H2_ is the permeability of H_2_ through membrane material (mol/m s Pa^0.5^), and, if Equation (4) is compared with Equation (5), then permeability can be expressed as

(6) $$Q_{H2}=\frac{DN_{b}X_{m}}{2}\sqrt{\frac{K_{H2}}{RT}}$$

However, permeability is typically expressed in terms of diffusivity (D) and solubility (S), as per equation: Q_H2_ = DS, therefore, considering Equation (6) and the latter definition the solubility (S) can be expressed as

(7) $$S=\frac{N_{b}X_{m}}{2}\sqrt{\frac{K_{H2}}{RT}}$$

Then, "Sieverts’ constant”, K_s_, (Pa^0.5^) as defined by Shu et al. [45,58], can be related to the adsorption equilibrium constant (K_H2_) reported in this study in Equations (4) and (5) as follows:

(8) $$\frac{1}{K_{s}}=\sqrt{\frac{K_{H2}}{RT}}$$

The Sieverts’ constant can also be theoretically calculated at a given temperature from the correlation (16) below, where $\Delta H_{H}^{o}$ and $\Delta S_{H}^{o}$ stand for the standards enthalpy and entropy for atomic hydrogen and it is obtained from the literature.

(9) $$K_{s}=76.8\;\exp\left(\frac{\Delta \bar{H_{H}^{o}}}{RT}-\frac{\Delta \bar{S_{H}^{o}}}{R}\right)$$

Additionally the “constant diffusivity”, D (m^2^/s), was calculated by employment of Equation (10) in which E_D_ = 4.5 E−7 m^2^/s and D_o_ = 24.1 kJ/mol (as given by Birnbaum and Wert [59]).

(10) $$D=D_{o}e^{-E_{D}/RT}$$

**Figure 3:**
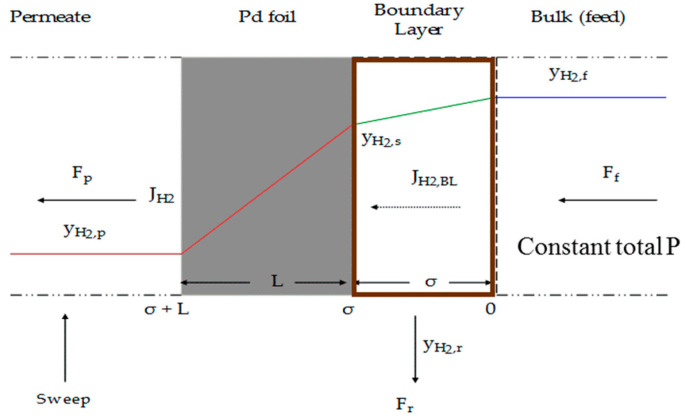
Schematic diagram showing concentration polarization in mixed feed.

When concentration polarization occurs, a layer adjacent to the surface at feed side is formed and hence hydrogen must initially diffuse through the boundary layer before reaching the membrane surface. Assuming a simple model where hydrogen is diffusing through a stagnant film of helium and if the diffusivity is assumed constant then, the steady state flux of molecular hydrogen through the boundary layer, J_H2,BL_ (mol/m^2^·s), can be described as follows [60]:

(11) $$J_{H2,\mathrm{BL}}=\frac{-D_{H2,\mathrm{He}}{\;P}_{f}}{\mathrm{RT}\sigma }\left(y_{H2,s}-y_{H2,f}\right)$$

where D_H2,He_ is the diffusivity of H_2_ in the feed mixture (m^2^/s), σ is the boundary layer thickness (m), P_f_ is the feed pressure (Pa), and y_H2,s_ and y_H2,f_ are the H_2_ mole fractions at the surface and in the bulk feed, respectively. Figure 3 shows the different layers through which hydrogen must penetrate through when, for example, a mixture feed of H_2_/He is introduced and hydrogen is only permeated through the membrane material. In this figure, F_f_, F_p_, and F_R_ are total molar flow rates (in mol/s) at feed, permeate, and retentate sides, respectively. The figure reflects the hydrogen molecule concentration or partial pressure distribution and helium is not shown but the boundary layer is the layer of stagnant helium limiting the hydrogen transport in the membrane.

The concentration polarization was experimentally analyzed; however, the validity of Sieverts’ Law was examined using Sieverts’ Law but the pressure exponent (*n*) to be unknown, i.e., an empirical fit parameter, as expressed in Equation (12) below

(12) $$J_{H2}=\frac{Q_{H2}}{L}\left(P_{H2,f}^{n}-P_{H2,p}^{n}\right)$$

The strong adsorption effect as well as the high temperature effect were theoretically evaluated using Equations (4), (6)–(10), and (12).

## 3. Materials and Methods

### 3.1. Materials

Palladium foils (25 µm thick and 99.9% purity) and palladium-silver foils (75:25 wt%, 25 µm thick and 99.9% purity) were provided by Alfa Aesar (Haverhill, MA, USA). The foils were mounted in stainless steel module to be introduced in the following section. Kalrez o-rings of 45.14 ± 0.46 O.D. and 42.52 ± 0.38 mm I.D, which lose its structural integrity at 589 K, was used to seal the module. Ultra-high purity (grade 5) of 99.999% hydrogen and pure helium (grade 5) with purity of 99.999% were provided from General Air (Exton, PA, USA). Helium was used as a leak detector, inert gas, and diluent in the mixed feed gas.

### 3.2. Methods

#### 3.2.1. Membrane Module Design

**Figure 4:**
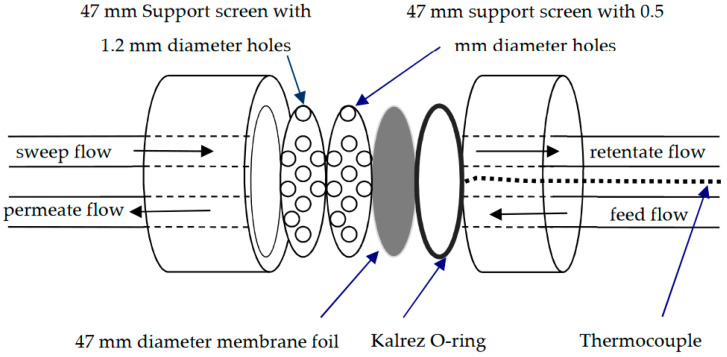
Schematic diagram of the used membrane module.

A membrane module was designed to insure high contact surface area and hence enhanced simulate of the realistic operating conditions. The module and inlet/outlet system were designed to fit a commercial filtration flange from Millipore with one port on each side, as illustrated in Figure 4.

The module has an outer diameter of 80 mm and an inner diameter of 47 mm. It can handle a maximum inlet pressure of up to 2.0 MPa and a maximum pressure drop across the flange of 355 kPa. The measurements were typically made with feed pressures of 101–214 kPa and pressure drops range 17–121 kPa. The module has two back pressure support screens with 0.5 mm and 1.5 mm diameter holes. The 45.14 ± 0.46 O.D. and 42.52 ± 0.38 mm I.D. Kalrez o-ring was used to seal the foil to the feed side of the module, and on the permeate side, the foil seals directly to the finer support screen. The effective inner diameter of the Kalrez o-ring was estimated to be ~40 mm. This matches the O.D. of the screen part of the finer support. Accordingly, a calculated permeation area of 12.57 cm^2^ was used for flux calculations.

The extent of mixing inside the module was characterized in a side experiment. The spreading of the peaks when the flow goes through the module indicates that mixing occurs in the module, and the composition inside the module on the feed side could assumed to be equal to the retentate composition rather than the feed composition.

#### 3.2.2. Membrane Foil Pretreatment

All Pd-based foils, used in this study, were rinsed with acetone and then placed in stagnant air environment inside a GC-8A oven (Shimadzu, Kyoto, Japan) and then activated, ex situ, at 673 K and ambient pressure for one hour. Once the foil was mounted in the holder, the module was purged with inert gas to remove any air inside before the run. The inert gas continued to flow while the module was heated to the required temperature, and hence the foil was also activated in situ in a flowing inert gas, from room temperature to the experiment temperature (i.e., a value of 473, 523, or 573 K).

#### 3.2.3. Experimental Setup

**Figure 5:**
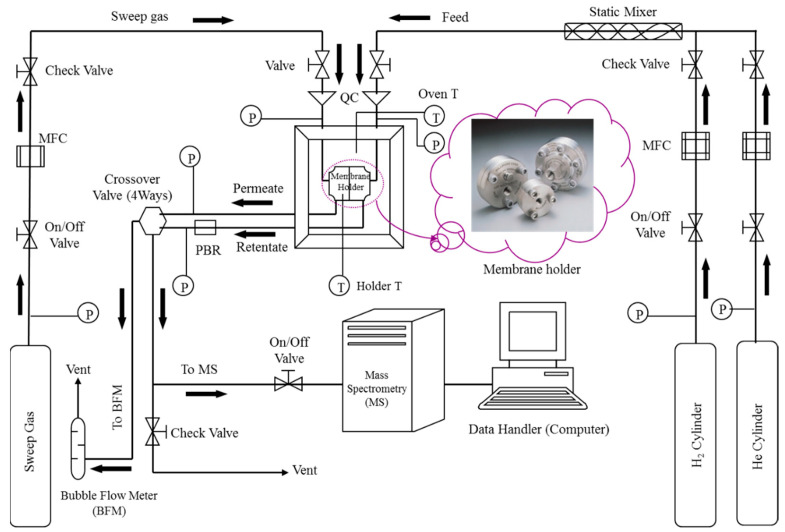
Schematic diagram of the experimental apparatus.

The used apparatus in H_2_ permeation studies is shown in Figure 5. It mainly consists of a membrane holder, gas cylinders, Brooks mass flow controllers (model 5850E, AK Ruurlo, The Netherlands), a (1–10–100 mL) bubble flow meter from Hewlett-Packard (Palo Alto, CA, USA), a flow system with Swagelok G.I. 60-psi range pressure gauges (Solon, OH, USA) two thermocouples from Omega (model HH21 type J, Norwalk, CT, USA), and a Prisma Quadrupole Mass Spectrometer model QMS 200 M2, purchased from Pfeiffer Vacuum (Wetzla, Germany). The membrane module was heated inside a GC-8A type gas chromatography oven (provided by GOW-MAC Instrument Company, Bethlehem, PA, USA) and the feed side pressures were established using a 0–100 psig (0–689 kPa) Swagelock KBP series diaphragm-sensing back pressure regulator (Cleveland, OH, USA). The feed and permeate pressures were measured with a 0–100 psia (0–689 kPa) CMM-121977 HEISE pressure gauge (Stratford, CT, USA). An in-line static mixer from Koflo (model 3/8-40c-4-6-2, Cary, IL, USA) was used to introduce the desired mixtures at feed side from pure gas cylinders of hydrogen and helium. All valves were supplied by Swagelok (Solon, OH, USA).

Hydrogen permeation through Pd and Pd-25% Ag foils was developed in the Millipore membrane module in a flexible set up with or without introducing a sweep gas to carry the permeated hydrogen either to a mass spectrometer for analysis or to the bubble flowmeter for measuring its flow rate, as indicated in Figure 5. The membrane holder, as earlier mentioned, has two inlets and two outlets that allow the flow across the feed and permeate surfaces. Helium was used to purge the system prior to permeation measurements, and also to sweep permeated hydrogen in some cases. The foils were 25 µm thick with 12.57 cm^2^ effective permeation area. The foil was activated (pretreated) and then mounted in the module. The helium purged air from the system and the module was heated to the run temperature, then the feed mixture was introduced to the module at the feed side to start the permeation experiments. Hydrogen permeation fluxes from mixture feeds were measured in the temperature range of 473–573 K and feed pressure range of ambient to 214 kPa for a helium mole percentage as low as 5% and as high as 83%, binary feeds. The feed flow rate was varied from 114 sccm to 485 sccm. Two foils of palladium (Pd-1 and Pd-2) and one foil of palladium-silver alloy (75% Pd-25% Ag foil) were used to obtain the results of this work.

#### 3.2.4. Theoretical Calculations

In this part of the study, the impact of various operating conditions on (n) was theoretically assessed. The modelling used Equations (4) throughout (12) to quantify the impact. To determine the effect of strong adsorption (high pressure limit) on ‘*n*’, hydrogen fluxes (J_H2_) were calculated by use of Equation (4) at various feed pressures (P_H2,f_) ranged from 1 to 136 atm, while permeate pressure was maintained at 1 atm. These calculated fluxes (J_H2_) were then substituted into Equation (12) using same operating conditions to calculate ‘*n*’ values as an empirical fit parameter. Similarly, the weak adsorption effect was modelled in the temperature range of 273–1273 K

## 4. Results and Discussion

The experimental studies analyzed hydrogen permeation fluxes from a mixed feed through two Pd (i.e., Pd-1 and Pd-2) and one Pd-Ag alloy 25 µm-thick foils at different operating conditions. The experimental studies on Pd-1 foil were achieved at an average temperature of 523 K to conceptually prove the effect of impurities and the driving force on mixed feed permeability; while for Pd-2 and Pd-Ag foils the mixed feed permeation fluxes were measured in the anticipated temperature range of 473–573 K. Additionally, the effect of strong adsorption (high pressure) and weak adsorption (high temperature) was also theoretically examined to verify the validity of Sieverts’ Law assumptions, and to analyze the realistic literature values of (*n*).

### 4.1. Permeability of H_2_ through Pure Palladium Foils

Two palladium foils (named Pd-1 and Pd-2) were used to study the effect of mixed feeds on hydrogen permeation. These foils had an effective permeation area of 12.57 cm^2^ and were activated as described in Section 3.

#### 4.1.1. Palladium Foil Pd-1

**Figure 6:**
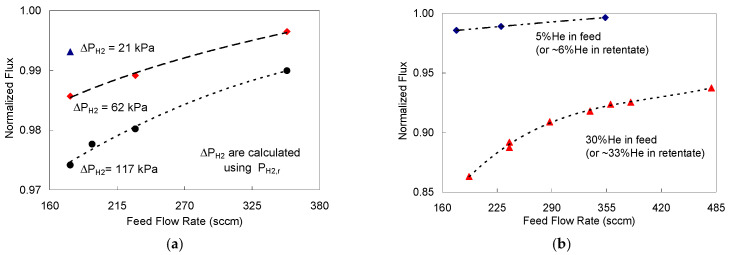
Hydrogen permeation fluxes through Pd-1 membrane foil at 523 K as a function of hydrogen pressure drop and feed flow rate: (**a**) for a 5% He feed stream with ∆P_H2_ of 21 kPa (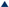), 62 kPa (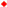), and 117 kPa (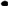); (**b**) for a 30% He feed stream (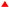) compared to 5% He feed stream (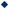) with ∆P_H2_ = 58 kPa and P_H2,f_ = 152 kPa.

The Pd-1 foil was mainly used to isothermally assess the effect of hydrogen partial pressure drop, the helium composition, and the feed flow rate on the degree of concentration polarization in mixed feeds permeation, at 523 K. A description of Pd-1 foil permeation operating conditions and the measurements are reported below (Table S1). Hydrogen fluxes from a 95% H_2_-5% He feed mixture at hydrogen partial pressure drops of 21, 62, and 117 kPa (P_H2,f_ basis) were normalized by the corresponding pure hydrogen fluxes through Pd-1 which were measured at similar conditions. For a leaner feed mixture (70% H_2_–30% He), the normalized H_2_ fluxes were calculated for a fixed hydrogen partial pressure drop of 62 kPa while varying the feed flow rate. Helium and hydrogen flows were adjusted to maintain the required constant hydrogen composition. The results are presented in Figure 6a for 5% He impurities in the feed while Figure 6b is for 30% He in feed.

The hydrogen fluxes for the 30% He were lower than those for the 5% He feed because of the stronger concentration polarization effect in the more dilute feed case, as expected [61]. Moreover, at feed flow rate of 180 sccm, the flux for 30% He feed was ≈12% lower than that of 5% He feed; while at higher feed flow rate of 355 sccm the percentage was cut in half (i.e., 6%), as can be observed from Figure 6b. The helium mole% in the retentate stream was calculated to be about 33% when ∆P_H2_ ≈ 58 kPa. As feed flow rate increased, the flux approached the pure hydrogen feed as the effect of concentration polarization decreased [17]. This behavior is concurred by the detailed results of 5% He in Figure 6a, at different pressure drops and feed flow rates.

**Figure 7:**
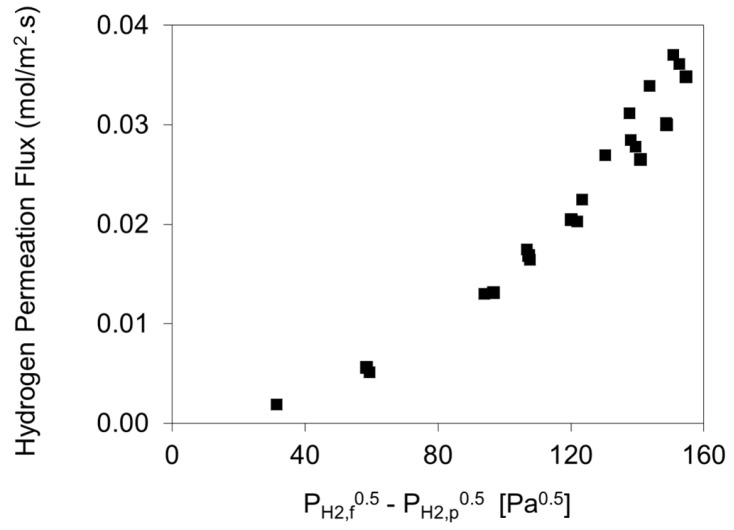
Mixed feed permeation through Pd-1 at 523 K for 8% to 83% He in feed balanced with hydrogen. Both sides of holder were at ambient pressure and helium used as sweep gas. Experimental runs were repeated as represented by the overlapped points.

Furthermore, hydrogen permeation from mixture feeds was also measured at 523 K maintaining ambient pressure on both sides of the membrane holder (feed and permeate). In this case, helium was used as a diluent in the feed and as a sweep on the permeate side. The helium sweep was maintained at 181 sccm, and on the feed side the helium composition was adjusted to vary from 8 to 83%. As shown in Figure 7, the H_2_ fluxes with the mixture feeds did not vary linearly with (P_H2,f_^0.5^–P_H2,p_^0.5^) as predicted by Sieverts’ Law.

To get a linear plot of hydrogen flux vs. (P_H2,f_*^n^*-P_H2,p_*^n^*) *n* value was empirically found to be close to 1 using feed compositions in the driving force and around 0.62 using retentate compositions in the driving force indicating the gap in hydrogen partial pressure between the bulk and the feed side membrane surface. Normally H_2_ permeation in the case of pure feeds is controlled by the feed and permeate pressures which are the same in bulk and close to the surface of the membrane, while in the case of mixture feeds, the more the He, or the non-permeable impurities in the feed, the greater the pressure drop in the gas film and accordingly lower driving force for hydrogen permeation [62].

#### 4.1.2. Palladium Foil Pd-2

**Figure 8:**
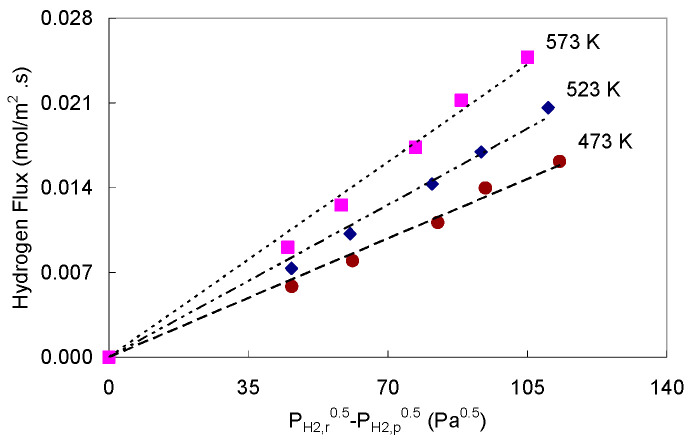
Hydrogen permeation fluxes, from a 13.5% He balanced with H_2_, through Pd-2 foil in the temperature range of 473–573 K and ΔP_H2,r_ of ~29, 40, 55, 64, and 79 kPa.

Table 1 summarizes the operating conditions of Pd-2 foil experiments for the permeation of 13.5% He in H_2_ mixed feed gas. The fluxes were measured at several temperatures and retentate-based hydrogen partial pressure drops while pressure at permeate side was kept as ambient pressure. The 0.5 power dependence of the fluxes are presented in Figure 8.

Clearly, the permeation did not follow Sieverts’ law as concentration polarization in the feed again affected the flux [30]. The helium composition in retentate varied from ~16.4 to 20.1 mol% as the pressure drop increased, at feed flow rate of 114 sccm; suggesting a more severe CP effect as more hydrogen gas permeated through the foil. To get these data to appear linear on a plot of flux vs. (Pn_H2,r_*^n^*-P_H2,p_*^n^*), *n* must be about 0.9, as an average value for the three temperatures. In this case, the activation energy for permeation determined using the Q values estimated simultaneously with *n* using expression (12) at various temperatures and estimated to be 8.12 kJ/mol. This value is less than the activation energy for pure hydrogen permeation through Pd-2, by ≈43%. The pre-exponential factor was 1.40 E-10 mol/m.s.Pa^0.9^, which as expected is also lower than that estimated for pure hydrogen permeation [26]. This illustrates that using Equation (12) when concentration polarization limits the permeation leads to under estimate of the permeability parameters.

### 4.2. Permeability of H_2_ through Palladium–Silver Foils

To verify the findings obtained for Pd-1 and Pd-2 foils, the hydrogen permeation experiments were repeated from a comparable He in H_2_ mixed feeds through 25 µm thick- 75% Pd-25% Ag foil (called Pd-Ag) at the operating conditions listed in Table 2. Hydrogen fluxes were obtained by adjusting the helium gas flow rate in the feed (i.e., increase or decrease). The experiments were repeated at different temperatures of 473, 523, and 573 K.

**Figure 9:**
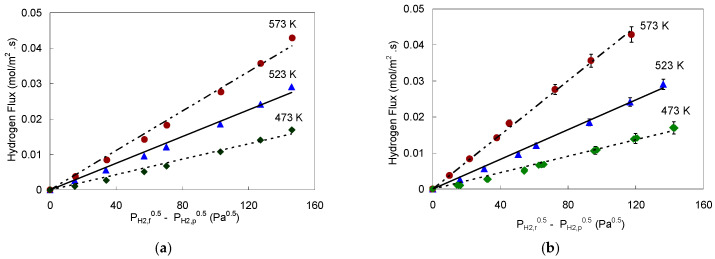
Hydrogen fluxes from a mixture of H_2_/He through Pd-Ag foil at 473, 523, and 573 K at P_f_ = 214 kPa and P_p_ = 93 kPa: (**a**) considering hydrogen partial pressure of the bulk feed (P_H2,f_); (**b**) assuming the hydrogen partial pressure in the retentate (P_H2,r_) for the composition. The straight lines to represent the best fit of Sieverts’ law if assumptions are valid.

In this study, it was observed that after each increase in helium flow rate in the feed, hydrogen flux at a given pressure was slowly decreasing. After each decrease in helium flow rate, the flux at a given pressure was slowly increasing. The steady state flux which is close to the average value of these two limits at a given pressure were used in the analysis. This method was used at all temperatures and H_2_/He compositions. The pressure dependence of the hydrogen fluxes, which clearly deviates from the 0.5-power Sieverts’ law, is shown in Figure 9a; where the partial pressure of H_2_ on the feed side was used assuming uniform composition.

While in Figure 9b, the flux vs. (P_H2,r_^0.5^–P_H2,p_^0.5^) was plotted using the retentate composition; a less dramatic deviation from Sieverts’ law was observed. The best fit of the data, presented in Figure 9a,b, was obtained when *n* ~ 1.4, when the feed composition was used, and *n* ~ 1.0, when the retentate composition was used, respectively. This suggests that diffusion of hydrogen through the bulk metal was not the rate limiting step in these measurements [63]. Using Equation (12) with the retentate composition as the feed composition and *n* = 1, an activation energy of 21.37 kJ/mol and a pre-exponential factor (Q_o_) of 2.53 E-10 mol/m.s.Pa were obtained. The values are not expected to be same as to those obtained from experiments in which Sieverts’ Law is valid [23]. Interestingly gas phase diffusion weakly depends on temperature compared to activated diffusion through membranes [62], accordingly the activation energy for these measurements is expected to be lower than that through the Pd-Ag membrane. Indeed, the estimated activation energy for transport through a boundary layer and then through the Pd-Ag membrane was found to be ≈18% lower than that for pure hydrogen transport alone through the same foil in the same temperature range. The values were obtained from the Arrhenius Plot using the results of Figure 9b.

### 4.3. Analyzing Sieverts’ Law Assumptions for the Mixed Feeds Permeation in Pd and Pd-Ag Foils

**Figure 10:**
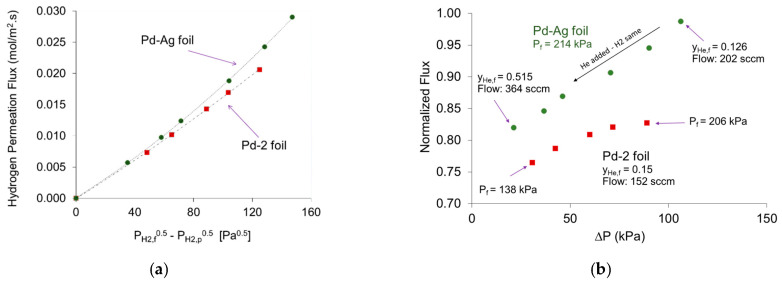
Hydrogen fluxes from a mixture of H_2_/He at 523 K through Pd-2 (in red symbols) and Pd-Ag (in green symbols) foils (**a**) as a function of P_H2_^0.5^ (Sieverts’ Law) for helium compositions of 0.15 and (0.126 to 0.515), respectively (**b**) as a normalized flux versus hydrogen pressure drop. Permeate pressure was 93 kPa.

As of the results presented earlier, it clear that hydrogen permeation from mixture feeds of hydrogen/helium through the Pd and Pd-Ag membrane foils in the current study deviated from Sievert’s Law at the conditions tabulated earlier. The comparative results of Pd-2 and Pd-Ag foils at 523 K were generated in Figure 10. As expected, H_2_ fluxes in Pd-Ag were higher than those of Pd-2 foil but, as previously indicated, both do not follow Sieverts’ 0.5-power law (Figure 10a). To better understand the concentration polarization behavior in the two foils, the normalized fluxes were considered in Figure 10b. H_2_ fluxes in Pd-2 foil were obtained by varying the feed pressure and maintaining the total feed flowrate at approximately 152 sccm while fluxes of Pd-Ag foil were obtained by adding more helium in the feed at constant total feed pressure of 214 kPa. Accordingly, for Pd-2 foil the increase in the feed pressure increased the driving force for hydrogen permeation, since the permeation process is fast a fluid phase layer was developed on the surface limiting the hydrogen flux and possibly led to surface saturation. On the other hand, for Pd-Ag foil, the added helium increased the formation of fluid phase layer but with relatively increased total feed flow rate, the CP effect was slightly minimized, and hence this explains the increase in the gap in Figure 10a as hydrogen pressure difference increased. These remarks support the results obtained for Pd-1 foil and illustrated in Figure 6.

Selective and fast hydrogen depletion through Pd-based membrane foils leads to accumulation of helium near the Pd surface, which causes a concentration gradient across a boundary layer in the feed [61]. Due to this phenomena, the partial pressure of hydrogen in the bulk feed is not equal to the partial pressure of hydrogen at the surface, which is a violation of one assumption in Sievert Law [17]. Hydrogen diffusion through the system can therefore be perfectly limited by the hydrogen diffusion through the boundary layer (fluid phase mass transfer) rather diffusion through the foil itself, which would normally lead to a linear dependence (i.e., *n* = 1) of flux on hydrogen partial pressures rather than the 0.5 exponent dependence of Sieverts’ law [23]. If both resistances are important, the exponent on the partial pressures can be between 0.5 and 1 [23]. Concentration polarization obviously also lowers the flux in addition to changing the pressure dependence of the flux, and improper analysis of fluxes measured under conditions where concentration polarization is completely or partly rate limiting leads to underestimates of the membrane permeability and its activation energy [17]. Furthermore, increasing the feed flow rate decreases the effect of concentration polarization. Considering the influence of the flow rate at the exit of the retentate side on H_2_ flux, a higher flow rate means more H_2_ being sent into the membrane surface, sweeping the other impurities and rendering a thinner H_2_ boundary layer along the membrane surface. This results in a larger H_2_ concentration gradient on the surface and thereby intensifying H_2_ flux [63]. Hence, the total feed flow rate should be considered when comparing hydrogen flux from feed mixture results, which is not necessary when comparing pure hydrogen permeation fluxes [13]. At higher partial pressure driving forces across the foil, concentration polarization is also more severe as would be expected.

## 5. Mathematical Modeling

Equations (4) and (12) were mainly used to model and to independently assess the strong and weak adsorption on validity of 0.5-power Sieverts’ law for the pure H_2_ permeation through 25 µm thick Pd foils. All parameters which were used in the model calculations are reported in Table 3.

### 5.1. Effect of High Pressure Limits (Strong Adsorption)

**Figure 11:**
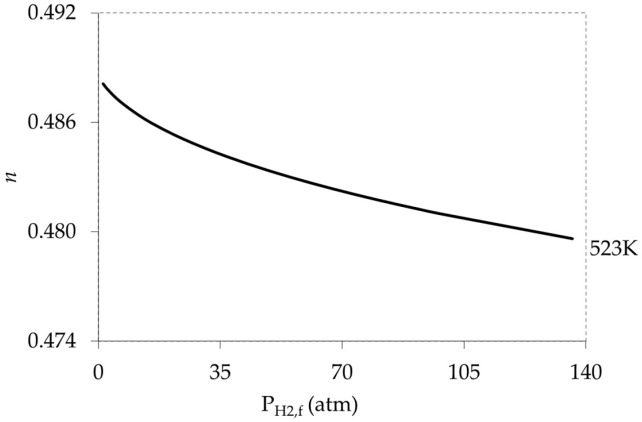
Modeling the effect of strong adsorption on pressure exponent (*n*) at 523 K.

The strong adsorption effect on hydrogen permeation and consequently on the power exponent (*n*) was assessed at 523 K for pure H_2_ permeation through Pd foil. The feed pressure (P_H2,f_) was varied from 101 to 13,736 kPa (i.e., 1 to 136 atm) with permeate side at ambient pressure (P_H2,p_). The results are presented in Figure 11. Additional temperature results in the range of 473–573 K are available in the supplementary information (Table S1).

The modelling results showed (*n*) values deviated from the 0.5 power. Clearly in Figure 11, as the P_H2_ increased the pressure exponent (*n*) decreased below 0.5. Theoretically, if P_H2_ approaches the infinity (∞) then the membrane will be saturated and hence *n* will approach zero, suggesting a permeation rate to be independent of P_H2_. Therefore, the very strong adsorption in the case of a very high applied pressure, hypothetically indicate that the assumption $\sqrt{\frac{KP}{RT}}\ll 1$ in deriving the Sieverts’ law is invalid.

### 5.2. Effect of Weak Adsorption (High Temperature Limits) on Pressure Exponent (n)

**Figure 12:**
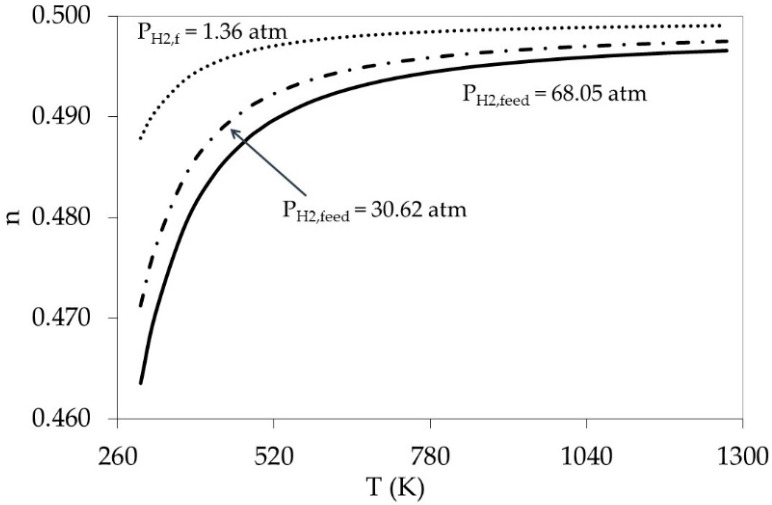
Modeling the effect of weak adsorption (high temperature) on hydrogen permeation rate and the corresponding (*n*) value for hydrogen partial pressures in the range of 1.36–30.62 atm.

Similarly, the effect of weak adsorption (high temperature) was modeled by varying the temperature from 298 to 1273 K. Three different feed pressures were chosen in the model, i.e., 1.36, 30.62, and 68.08 atm. The permeate side was kept at ambient conditions. The temperature dependency, as per model used, is reported in Figure 12. An increase of absolute temperature by ≈3 times resulted in an increase of *n* values by 7% towards the 0.5 value. The weak adsorption supports the Sieverts’ law validity because it validates the assumption $\sqrt{\frac{KP}{RT}}\ll 1$.

### 5.3. Combining the Effect of Pressure and Temperature in the Model

All results in Figure 11 and Figure 12 were obtained assuming low pressure at permeate side, and also individually addressed the effect of each parameter on (*n*). For example, an increase in feed pressure by ≈130 atm caused a decline of (*n*) value by only 2%. While a decrease of the temperature by 3 times lowered the (*n*) value by 7%. Furthermore, a simultaneous increase of pressure accompanied by a decrease of temperature resulted in a 30–50% more effect on (*n*) because the assumption $\sqrt{\frac{KP}{RT}}\ll 1$ is strongly not valid.

## 6. Explaining the Literature (n) Values and the Future Work Direction

The effect of high sorption coverage, i.e., high pressure or high adsorption equilibrium constant, as well as the concentration polarization were previously investigated. Concentration polarization resulted in pressure exponent (*n*) values between 0.5 and 1.0, a high sorption theoretically assessed and produced (*n*) values below 0.5. These effects were independently addressed and their impacts were quantified. If combined, the degree of the deviation from Sieverts’ law will probably depend on the contribution of each step in the permeation process.

Table 4 summarizes the membranes features and the operating conditions for some realistic studies, in which (*n*) was treated as an empirical fit parameter and its value varied from 0.5 to 1.41, as follows: (i) supported or non-supported Pd as thin as 0.5 µm and as thick as 1000 µm, (ii) temperature range of 623–1219 K, (iii) pure feed gas or mixed feed with up to 80% He, (iv) feed pressure in the range of ≈1–27 atm, and (v) permeate pressure either as vacuum or 1 atm (sweep gas). The (*n*) values were qualitatively interpreted and possible explanations were provided according to current study outcomes.

Hurlbert and Konecny [66] reported an (*n*) value of 0.68 for pure H_2_ permeation through 10–150 µm (relatively) thick Pd membranes with relatively high P_H2,f_ = 7 atm and T > 623 K and permeate side at vacuum. This value can be justified by the combined effect of thick membrane (*n* = 0.5), high T (*n*→0.5), high P_f_ (*n* < 0.5), and desorption limiting (*n* ≈ 1). Moreover, Katsuta and coworkers [67] assessed hydrogen permeation through a very thick Pd membrane at operating conditions favored the 0.5 power. Furthermore, Morreale et al. (2003) [24] investigated the mixed feed (10% He balanced with H_2_) permeation for very thickness Pd in a relatively high temperature and pressure which resulted in a pressure exponent value between 0.5 and 1.0. Interestingly Gielens and coworkers [25] conducted two studies one with support and one without, however, in both 80% He–20% H_2_ mixture was introduced to a very thin palladium at relatively high temperature. For the non-supported Pd the strong concentration polarization gave *n* ≈ 1, while in the supported-Pd membrane the contribution was cumulative (CP → n = 1, support → J_H2_,_support_ α P^2^ → n = 2, high T → n = 0.5) so *n* was estimated to be ~1.4 [68]. Most recently Chen and coworkers [61] reported exponent values of 0.5 for very thin tubular Pd membrane at relatively high temperature and moderate pressure drop. When 25% N_2_ balanced with hydrogen was introduced to the system a severe CP effect was observed leading to an (*n*) value of 1.0, as expected.

The above qualitative analyses of *n* values in Table 4 clearly support the findings of this work. The values obtained by qualitative analyses were close enough to the literature-reported values. However, this study did not take into account the Pd surface contaminants [69,70,71] or the membrane activation [72] to physically or chemically block the surface. Nevertheless, the better understanding of the assumptions in Sieverts’ Law enabled the authors to explain some of the literature values for similar conditions, such the nature of the feed gas, operating conditions, and nature of the membrane (thickness, dense or composite). Additionally, Suzuki and coworkers reviewed various methods for consistent analysis of hydrogen permeability [73].

**Figure 13:**
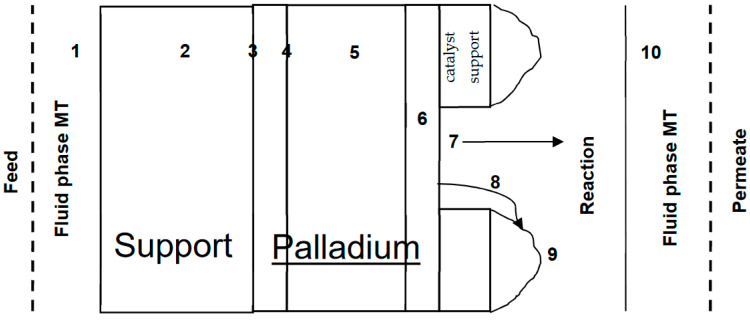
Schematic diagram of resistances to hydrogen transport through a catalytic Pd-based membrane reactor, with supported catalyst.

The limited applicability of the current approach suggests to better understand the H_2_ transport in the palladium-based catalytic membrane reactors and consequently the Sieverts’ Law assumptions. This requires a general transport model to incorporate all possible mass transfer resistances [74], in addition to the reaction. The incorporation of general model in assessing the assumptions of Sieverts’ Law and quantify the role of each effect (i.e., mixed feed, support, thickness, catalyst, T, P, etc.) The general model aims at numerically determining the pressure exponent (n) values. Figure 13 shows the possible resistances in a typical Pd-membrane reactor; as follows: (1) fluid phase mass transfer, (2) flow through support, (3) H_2_ dissociative adsorption on surface, (4) H from surface to inside Pd, (5) H diffusion in Pd, (6) H from bulk to surface, (7) H atoms re-combinative desorption, (8) H atom spill-over on catalyst, (9) surface reaction, and (10) unreacted or produced H_2_ flow through fluid phase mass transfer. This proposed transport maybe slightly different if hydrogenation or dehydrogenation reactions involved, or there is a need for support on both sides, or the nature and stability of the catalyst (i.e., metal segregation), and finally the shape of the module (disk, sheet, tube, hollow fiber, etc.).

## 7. Conclusions

Palladium membranes can be effectively employed inside reactors, and most recently for hydrogen production from fossil fuels or from bio-wastes. The use of Sieverts’ law with 0.5 exponent in such complicated system would either underestimate or overestimate the hydrogen flux. Hence, some of the assumptions used in deriving Sieverts’ Law were assessed in this study, either experimentally or by means of a simple model, to describe the reported literature pressure exponent (*n*) in comparable studies. The assumptions of our interest are: (i) diffusion of H through Pd metal is rate limiting, (ii) there is no concentration gradient at the feed side, (iii) no species other than H_2_ on surface, and (iv) weak adsorption behavior on surface. Hydrogen permeation studies from a mixture feed through Pd-1, Pd-2, and Pd-Ag foils at different temperatures and pressure drops resulted in a decline of H_2_ flux suggesting formation of an impermeable gas close to the membrane surface (CP effect). The (*n*) value of 1.0, which was obtained from the experimental data, suggests that concentration polarization to be dominant and hence assumption (i) probably invalid. The pressure exponent (*n*) of 0.9 for Pd-2 foil fluxes confirms a partial contribution of mixed feed effect on the validity of assumption (i), as rate limiting step. Moreover, the Pd-1 studies stressed on importance of the feed flow rate to minimize the CP effect, a 7% increase was observed in the normalized flux for 30% He in feed when total feed flow rate was doubled. This explains that the actual driving force for the permeation is lower than the theoretical value, i.e., P_H2,f_ > P_H2,surface_, and therefore, probably assumption (ii) in Sieverts’ law is not anymore valid. Furthermore, the use of mixed feed with CP limitations may also affect the assumption (iii), as helium physically stays on the foil surface. A stronger effect of this assumption can be observed if contaminants like carbon chemically adsorbs on the surface, but in our study this was minimized or eliminated when membranes foil were ex situ activated at 673 K. Lastly, assumption (iv) was examined at high adsorption coverage, i.e., at high pressure and low temperature, using a simple model assuming Langmuir adsorption with temperature dependent adsorption equilibrium coefficient. Mathematical modelling confirmed that high pressures or low temperatures could lead to *n* < 0.5 but based on the literature values normally *n* is ≥0.5, which may suggest that the two effects are present but marginalized with the stronger impact of the other steps. The experiments and modelling in this study offer possible reasons explaining why experimentally fit value of the exponent, *n*, deviate from 0.5. A more general model including concentration polarization and high adsorption coverage effects on hydrogen transport in metal-based catalytic membrane reactor was presented, accounting for the catalytic multi-layered porous support.

## Figures and Tables

**Table 1:** Operation conditions for H_2_ in 25 µm thick Pd-2 membrane foil permeability studies.

Temperature (K)	Partial Pressure Drop (kPa)	Feed Mixture % He in H_2_	Feed Flow Rate (sccm)
473, 523, 573	~29, 40, 55, 64, 79	~13.5%	114

**Table 2:** Operation conditions for 25 µm-thick (75–25%) Pd-Ag membrane foil.

Temperature (K)	Feed Mixture % He in H_2_	Feed Flow Rate (sccm)	Feed Pressure (kPa)	H_2_ Feed Flow Rate (sccm)	Permeate Pressure (kPa)
473, 523, 573	10–57%	15–172	214	129	93

**Table 3:** Parameter values used in model calculations.

Parameter	Value	Units	References
Q_o_	9.29 × 10^−8^	mol/m s Pa^0.5^	(Current study)
E_p_	11.7	kJ/mol	(Current study)
D_o_	4.5 × 10^−7^	m^2^/s	Birnbaum and Wert(1972) [59]
E_D_	24.1	kJ/mol	Birnbaum and Wert (1972) [59]
$\Delta \bar{H_{H}^{o}}$	−8.4	kJ/mol	Holleck (1991) [64]
$\Delta \bar{S_{H}^{o}}$	−48.7	J/mol K	Holleck (1991) [64]
N_b_	1.13 × 10^5^	mol Pd/ m^3^	Ward and Dao (1999) [22]
X_m_	0.65	mol H/mol Pd	Smirnov and Gol’tsov(1988) [65]
R	8.314	J/mol K	(Gas constant)

**Table 4:** Literature pressure exponent (*n*) values for hydrogen permeation through supported and unsupported Pd membranes from pure and mixture feeds.

References	Hurlbert and Konecny [66]	Katsuta et al. [67]	Morreale et al. [24]	Gielens et al. [25]	Gielens et al. [25]	Chen et al. [61]	Chen et al. [61]
Parameters							
L (µm)	10–150	940	1000	0.9	0.5	6.93	6.63
	no support	no support	no support	no support	with support	Tubular	Tubular
	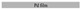	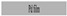	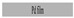	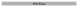	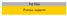	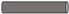	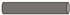
T (K)	623–773	769–1219	623–1173	623–873	623–873	623	623
Feed gas	H_2_ 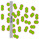	H_2_ 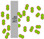	90% H_2_–10% He 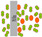	20% H_2_–80% He 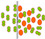	20% H_2_–80% He 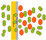	H_2_	75% H_2_–25% N_2_
P_total,f_ (kPa)	101–710	101	101–2760	101–505	101–505	101–202	303–506
P_H2,f_ (kPa)	101–710	101	91–2480	20–101	20–101	101–202	303–506
P _total,p_ (kPa)	Vacuum	Vacuum	118	101	101	101	101
Permeate condition	Vacuum	Vacuum	Ar sweep	N_2_ sweep	N_2_ sweep	N_2_ sweep	N_2_ sweep
*n*	0.68	0.5	0.62	0.5–1.04 **	0.58–1.41 **	0.5	1.0
Possible justification of (*n*)	L-Thick Pd *** Pure H_2_ (*n* ≈ 0.5), high P (*n* < 0.5), High T (*n* ~ 0.5), high ΔP_H2_ desorption rate limiting (*n* ≈ 1) → Avg. (*n*) = 0.7	H-Thick Pd Pure H_2_ (*n* = 0.5), (low P and high T) (*n* = 0.5), low ΔP_H2_ → Sieverts’ Law (*n* = 0.5)	Thick Pd (*n* = 0.5), with lower CP * effect at feed (*n* ~ 0.8), Very high P (*n* < 0.5), high T (*n* ~ 0.5), → Avg. (*n*) = 0.63	Thin Pd Strong mixed effect (CP *) and sweep (*n* = 1), High T (*n* ~ 0.5), moderate P (*n* ~ 0.5), → Very thin membrane with strong CP (*n* = 1)	Thin Pd Strong mixed feed or CP * (*n* = 1), high T (*n* ~ 0.5), Viscous flow in support flux α P_H2_^2^ (*n* = 2), moderate P (*n* ~ 0.5) → Avg. (*n*) = 1.38	Thin Pd Pure H_2_ low P (*n* = 0.5), High T (*n* = 0.5), → Avg. (*n* ~ 0.5)	Thin Pd Strong mixed feed or CP * (*n* = 1), low P (*n* = 0.5), High T (*n* = 0.5), → mass transfer in fluid phase is the limiting step (*n* ~1.0)

* CP is an abbreviation for concentration polarization. ** Upper limit was evaluated. *** L-Thick Pd means relatively thick Pd and H-Thick Pd refers to a very thick film.

## Data Availability

Not applicable.

## Supplementary Materials

The following are available online at https://www.mdpi.com/article/10.3390/membranes11100778/s1, Figure S1: Modeling the effect of strong adsorption (high P) on pressure exponent (*n*) for temperature range of 473-573 K. Table S1: Operation conditions for H2 in 25 µm thick Pd-1 membrane foil permeability studies.
