# Supplementary material for: Assessment of Sieverts Law Assumptions and ‘n’ Values in Palladium Membranes: Experimental and Theoretical Analyses
Source: Membranes (Basel). 2021 Oct 12;11(10):778. doi: 10.3390/membranes11100778 (PMC8540459; doi:10.3390/membranes11100778)
Supplement: Supplementary file 1 [file membranes-11-00778-s001.zip › membranes-1384139-supplementary.pdf]

membranes

MDPI

Supplementary Material

Assessment of Sieverts Law assumptions and '*n*' Values in Palladium Membranes: Experimental and Theoretical Analyses

Abdulrahman Alraeesi [1,*] and Tracy Gardner [2]

[1] Chemical and Petroleum Engineering Department, United Arab Emirates University, P.O Box 15551, Al Ain, United Arab Emirates; a.alraeesi@uaeu.ac.ae
[2] Chemical and Biological Engineering Department, Colorado School of Mines, Golden, CO 80401, USA; tgardner@mines.edu
* Correspondence: a.alraeesi@uaeu.ac.ae

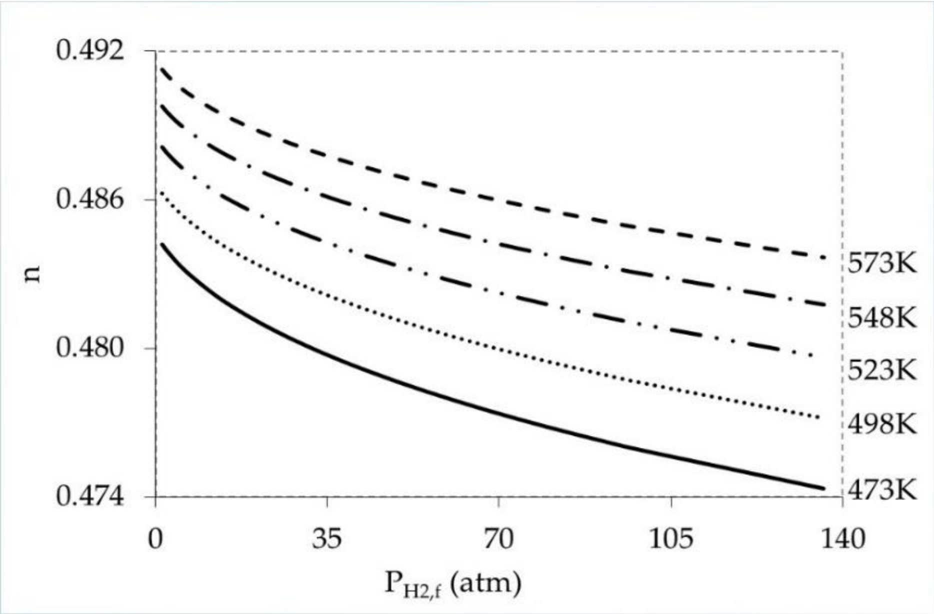

Figure S1. Modeling the effect of strong adsorption (high P) on pressure exponent (*n*) for temperature range of 473-573 K.

Table S1. Operation conditions for H_2 in 25 μm thick Pd-1 membrane foil permeability studies.

Temperature (K)	Partial pressure drop (kPa)	Feed mixture % He in H_2	Feed pressure (kPa)	Permeate pressure (kPa)
523	21, 62, 117	5–83	221	93
